# Warming Decreases Bioconversion of Polyunsaturated Fatty Acids in Chironomid Larvae Maintained on Cyanobacterium *Microcystis*

**DOI:** 10.3390/biom11091326

**Published:** 2021-09-07

**Authors:** Ursula Strandberg, Timo Ilo, Jarkko Akkanen, Paula Kankaala

**Affiliations:** Department of Environmental and Biological Sciences, University of Eastern Finland, 80101 Joensuu, Finland; timo.ilo@uef.fi (T.I.); jarkko.akkanen@uef.fi (J.A.); paula.kankaala@uef.fi (P.K.)

**Keywords:** ARA, climate change, *Chironomus riparius*, desaturation, EPA

## Abstract

Cyanobacteria dominance and warming have been suggested to decrease the production of polyunsaturated fatty acids (PUFA) in freshwater ecosystems. Physiological adaptations of poikilothermic animals to higher temperatures may further decrease PUFA levels in aquatic food webs. We conducted diet manipulation experiments to investigate the combined effects of dietary PUFA and warming on the proportions of eicosapentaenoic acid (EPA) and arachidonic acid (ARA) in *Chironomus riparius*. The experimental diet consisted of a nontoxic cyanobacterium *Microcystis*, which contained C_20_ PUFA: 20:3n-3, 20:4n-3, and 20:3n-6, but no EPA or ARA. Additionally, we used TetraMin^®^ fish flakes as a control treatment. A temperature increase from 20 °C to 25 °C decreased the proportion of n-3 C_20_ PUFA and the n-3/n-6 ratio in *Microcystis*. Diet manipulation experiments indicated that *Chironomus* desaturated dietary C_20_ precursors to EPA and ARA, but warming decreased this bioconversion and resulted in lower levels of EPA and ARA in *Chironomus*. Warming did not alter the proportions of EPA and ARA in *Chironomus* larvae if these PUFA were readily available in the diet (TetraMin^®^ control treatment). In conclusion, warming and cyanobacteria dominance may decrease the production and trophic transfer of physiologically important PUFA in freshwaters by (1) decreasing the n-3/n-6 ratio and the abundance of n-3 C_20_ precursors in *Microcystis*, and (2) decreasing the bioconversion of n-3 and n-6 C_20_ precursors to EPA and ARA in chironomids. These changes may have cascading effects throughout the food web and decrease the content of EPA in fish, potentially affecting its availability to humans.

## 1. Introduction

Freshwater ecosystems are particularly susceptible to global warming because lake chemistry and hydrology are highly climate-dependent, and aquatic ecosystems are already exposed to numerous anthropological stressors, such as increased loading of nutrients, leading to eutrophication of lakes and rivers [1,2]. Increased algal and cyanobacterial blooms threaten the functioning of aquatic ecosystems, fisheries, and human health [3,4]. Increased cyanobacteria dominance and warming have been suggested to decrease the basal production of n-3 and n-6 polyunsaturated fatty acids (n-3 and n-6 PUFA) [5,6]. The decreased basal production of n-3 and n-6 PUFA is predominantly caused by changes in the phytoplankton community structure because the composition of PUFA in algae and cyanobacteria is phylogenetically determined [7,8]. Cyanobacteria do not contain highly unsaturated PUFA, such as arachidonic acid (ARA), eicosapentaenoic acid (EPA), or docosahexaenoic acid (DHA) [9], which are considered physiologically important for most animals. Additionally, laboratory experiments indicated that warming alters the fatty acid composition in algae and cyanobacteria [10,11]. In general, the proportion of PUFA and the n-3/n-6 ratio in phytoplankton decrease with increasing temperature [10,11].

Trophic transfer and trophic upgrading of PUFA are highly important in determining the availability of PUFA to upper trophic level consumers, such as fish. Trophic upgrading refers to mechanisms in which a consumer “improves” the dietary fatty acid composition by (1) selectively retaining important PUFA in their tissues or (2) modifying dietary PUFA to physiologically more active forms, typically via chain elongation and/or desaturation [12,13]. Generally, the abundance of C_20_ and/or C_22_ PUFA increases with each trophic step [14], but exceptions are common. For instance, retroconversion of C_22_ PUFA to C_20_ PUFA has been detected in, for example, cladocerans and chironomids [15,16]. Chironomids (Diptera: Chironomidae) are nonbiting midges whose life cycle includes aquatic egg, larval and pupal stages, and an aerial adult stage [17]. They are opportunistic omnivorous feeders with wide ecological tolerance and a short life cycle, enabling them to thrive in diverse habitats, in both the lentic and lotic environments [18]. Chironomids are a dominant group in freshwater ecosystems, often accounting for more than 50% of the macroinvertebrate community in lakes [19]. Chironomids are ectotherms; thus, their body temperature reflects that of the environment and ambient temperature drives physiological processes. For instance, the developmental rates increase with ambient temperature until a taxaspecific limit is reached [20,21,22]. Additionally, the size and fecundity of females have been suggested to decrease at high temperatures [21]. In addition to temperature, the quantity and quality of diet, specifically PUFA availability, have been linked to the developmental rate and growth of chironomids [23,24].

**Figure 1:**
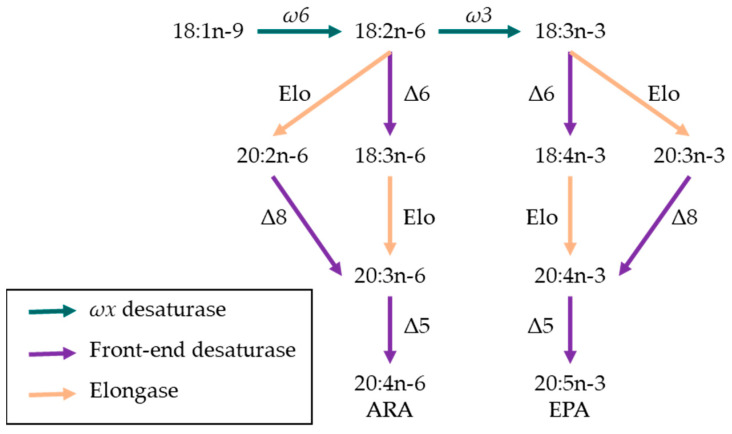
Simplified depiction of potential biosynthetic pathways of polyunsaturated fatty acids discussed in the current study (modified from Monroig and Kabeya 2018). Δx and *ωx* desaturases refer to the position of the added double bond; position of the added double bond is marked either from the methyl end (*ω* desaturases) or from the carboxyl end (Δ desaturases, also called front-end desaturases) of the acyl chain. Elongation reactions are marked with “Elo”.

We have previously shown that *Chironomus riparius* (hereinafter referred to as *Chironomus*) maintained on cyanobacterium *Microcystis* have surprisingly high levels of EPA and ARA, considering that these PUFA are not found in *Microcystis* [16]. Dietary C_18_ precursors alone could not explain the higher-than-expected ARA and/or EPA levels in *Chironomus* [16]. We concluded that most likely the C_20_ precursors in *Microcystis*, i.e., 20:3n-6 and 20:4n-3, were Δ5-desaturated to ARA and EPA, respectively (Figure 1). Theoretically, *Chironomus* could also express Δ8-desaturase activity, i.e., desaturation of dietary 20:3n-3, which is also found in *Microcystis*, to 20:4n-3, after which 20:4n-3 may be Δ5-desaturated to EPA [25]. The fatty acid modification pathways in invertebrates differ from those of mammals [25,26], and Δ8-desaturase activity has been suggested in certain teleost fish, molluscs, and annelids [25,27]. However, we are not aware of any study showing Δ8-desaturase activity in chironomids, but this warrants further study, and thus we also considered 20:3n-3 as a potential precursor for EPA.

The aim of this study was to evaluate the effects of C_20_ precursor abundance in the diet and ambient temperature on the proportions of ARA and EPA in *Chironomus* larvae. We manipulated the cellular fatty acid composition in *Microcystis* by culturing it at different temperatures, after which *Microcystis* were fed to *Chironomus* larvae maintained at 20 °C and 25 °C. We hypothesized that warming will decrease the proportion of C_20_ precursors and the n-3/n-6 ratio in *Microcystis*, as shown in a previous study [11]. We also hypothesized that A) the abundance of C_20_ precursors in the diet affects the proportions of ARA and EPA in *Chironomus* larvae, and B) warming decreases the proportion of ARA and EPA in *Chironomus* larvae maintained on *Microcystis* because of downregulated Δ5-desaturase activity at higher temperatures, as shown for a cyclopoid copepod, *Paracyclopina nana* [28].

## 2. Materials and Methods

We conducted diet manipulation experiments on *Chironomus* larvae at different temperatures. The experiments were conducted in a temperature-controlled room at 20 °C and 25 °C. We had four diet treatments: two experimental diets and two controls. The experimental diets consisted of fresh nontoxic cyanobacteria *Microcystis*, which had been cultured at 20 °C or 25 °C, hereinafter referred to as *Micro20* and *Micro25*, respectively. The cultures were maintained at these temperatures for at least one month prior to the experiments. In addition to the *Microcystis* diet treatments, we had a positive control (TetraMin^®^ fish food, Tetrawerke, Melle, Germany) and a negative control (no food). We used natural sediment in the experiments, thus the negative control was included to exclude possible sediment effects on *Chironomus* fatty acids. The sediment was collected on 4 June 2019 from Lake Höytiäinen (coordinates 62°03.533′ N, 26°08.167′ E) and sieved through a 1 mm sieve to remove animals and larger particles and stored at 4 °C. Homogenized sediment was portioned to 400 mL beakers after which the beakers were filled with artificial freshwater (Ca + Mg hardness 0.5 mmol^−1^, pH 6.6) and left to settle for 2 days. The sediment to water ratio was 1:4.

We used newly hatched (less than 48 h) first instar larvae in the experiments. The larvae were obtained from cultures maintained at ~20 °C at the University of Eastern Finland, Joensuu. Fresh egg clutches were collected and left to hatch in small beakers at the experimental temperatures, i.e., either at 20 °C or at 25 °C. Temperatures within the range of 15–27 °C have not been noted to affect the survival of *Chironomus riparius* larvae [22]. Each treatment had five replicate beakers, and each beaker had 10 larvae. Beakers were continuously aerated. Larvae were fed every other day, and diet was adjusted to ~0.4 mg C larva^−1^ day^−1^. Particles were allowed to set for an hour before continuing with the aeration. The experiments lasted nine days and on the fifth day, about half of the water in the beakers was siphoned and replaced with new artificial freshwater. The experiments were conducted at 20 °C and 25 °C with 16:8 h light:dark cycle. During the experiment, temperature variation was less than ±1 °C, dissolved oxygen saturation was greater than 60%, and ammonium (NH_4_^+^) content did not rise above 18 mg L^−1^, i.e., NH_4_^+^ content was below a harmful level [29,30]. At the end of the experiment, the pH in the *Micro20* and *Micro25* treatments was 6.8–7.1, thus within the OECD guidelines (pH 6–9) [29]. In the positive control (TetraMin diet), the pH was 5.6–6 and in the negative control (no food) 5.5–6. However, we did not observe negative effects on larvae growth or survival in the positive control, and predictably all larvae died in the negative control.

At the end of the experiment, the larvae were sieved from the sediment, counted and stored at −80 °C. All the individuals were used for the fatty acid analyses and thus we did not measure larval length or estimate the instar. Samples were lyophilized and weighed. The sample dry weight was 0.6–2.6 mg. Samples were extracted twice with chloroform: methanol (2:1) [31], and 10 µg of free fatty acid 23:0 was added as an internal standard. Extracted lipids were transmethylated at 90 °C for 90 min. using 1% sulfuric acid in methanol as a catalyst, see method details in Strandberg et al. [16]. Fatty acid samples were analyzed with an Agilent 6890N gas chromatogram equipped with an Agilent 5973N mass selective detector (Agilent Technologies, Santa Clara, CA, USA). The column was a DB-23 (Agilent length 60 m, inner diameter 0.25 mm, film thickness 0.15 µm). We used helium as a carrier gas with an average velocity of 27 cm s^−1^. The initial oven temperature was 50 °C, which was held for 1 min, after which the temperature was raised by 15 °C min^−1^ until 150 °C, and then by 0.5 °C min^−1^ until the oven temperature was 170 °C, and finally by 2 °C min^−1^ until the final temperature of 230 °C was reached. We used mass spectra and reference standard GLC-538 (Nu-check prep.) for peak identification. GLC-538 was also used as a calibration standard for the quantification of fatty acids. Fatty acids were presented as weight % of total fatty acids. Total fatty acids were presented as micrograms of fatty acid per milligram dry weight (µg mg^−1^ DW). Larval weights were presented as mg DW ind^−1^.

We used two-way ANOVA to test the differences in the fatty acid response variables (EPA w% or ARA w%), total fatty acid content and dry weight in *Chironomus* larvae, using ‘Diet’ and ‘Temperature’ as independent variables. ‘Diet’ included three levels: *Micro20*, *Micro25*, and TetraMin^®^ and ‘Temperature’ two levels: 20 °C and 25 °C. We used 0.05 as the alpha level and Type III sum of squares. We used Levene’s test to investigate the homogeneity of variances in the data. Larval dry weight, total FA content, and ARA w% had equal variances. The EPA w% was the arcsine square root transformed to achieve equal variances. Statistical analyses were performed with IBM SPSS Statistics 27.

## 3. Results

The *Microcystis* diet contained C_20_ precursors but not EPA or ARA, while the TetraMin^®^ control diet contained EPA and ARA (Table 1). The culture temperature altered the fatty acid profile of *Microcystis*. The proportion of 20:3n-6, which is a C_20_ precursor fatty acid for ARA, increased from about 0.35 w% in *Micro20* to 0.71 w% in *Micro25*. The proportion of 20:3n-3 and 20:4n-3, potential C_20_ precursor fatty acids for EPA, declined from 0.84 w% to 0.19 w% and from 1.93 w% to 0.21 w%, respectively, when *Microcystis* was cultured at the higher temperature. Moreover, the proportion of n-6 C_18_ PUFA increased and n-3 C_18_ PUFA decreased in *Microcystis* at the higher temperature (Table 1). Overall, warming decreased the proportion of n-3 PUFA and increased the proportion of n-6 PUFA in *Microcystis* (Table 1). Consequently, the n-3/n-6 ratio in *Microcystis* declined from 2.48 to 0.47 as the culture temperature increased from 20 °C to 25 °C. Subsequently, the *Micro20* diet can be characterized as a higher quality diet than *Micro25*, in terms of the abundance of n-3 C_20_ precursors and the n-3/n-6 ratio.

### 3.1. EPA and ARA in Chironomus

**Figure 2:**
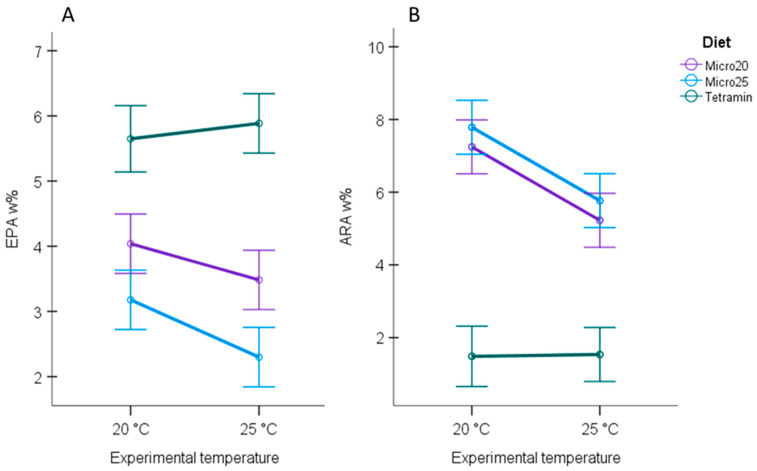
Estimated marginal means for (**A**) EPA w% and (**B**) ARA w% in *Chironomus* fed with either *Micro20*, *Micro25*, or TetraMin^®^ (control) and maintained at two different experimental temperatures: 20 °C and 25 °C. Error bars represent 95% confidence intervals. The mean EPA w% values differed between the *Microcystis* diet treatments (*Micro20* and *Micro25*). The mean EPA w% decreased with increasing temperature in both diet treatments, but the decrease was statistically significant only for the *Micro25* diet. The mean ARA w% in *Chironomus* did not differ between *Micro20* and *Micro25* diets, and the temperature significantly deceased ARA w% in both diet treatments. Temperature did not affect the EPA w% and ARA w% in *Chironomus* fed with TetraMin^®^.

Diet and temperature significantly affected the proportions of EPA and ARA in *Chironomus* (Table 2). Diet could explain ~83% of the variation in EPA w% and ~81% of the variation in ARA w% (Table 2). Temperature could explain ~2% of the variation in EPA w% (Table 2) and ~7% of the variation in ARA w% (Table 2). The highest proportion of EPA was found in *Chironomus* fed with TetraMin^®^, regardless of the experimental temperature (Figure 2A). In contrast, the proportions of ARA were lower in *Chironomus* fed with TetraMin^®^ than in *Chironomus* fed with either *Micro20* or *Micro25*, but the ARA levels did not differ between *Micro20* and *Micro25* diets. Warming did not affect the EPA w% and ARA w% in *Chironomus* fed with TetraMin^®^ but decreased the proportions of EPA and ARA in *Chironomus* fed with *Microcystis* (ESM Tables S1 and S2). In *Micro20* and *Micro25* diet treatments, warming significantly decreased the proportion of ARA in *Chironomus* larvae from about 7.2–7.8 w% to 5.2–5.8 w% when the experimental temperature increased from 20 °C to 25 °C (ESM Table S1). In the *Micro20* treatment, the EPA w% in *Chironomus* declined from 4.0 w% to 3.5 w%, but the decline was not statistically significant (*p* = 0.098, ESM Table S2), whereas in the *Micro25* treatment the temperature-induced decline in EPA from 3.2 w% to 2.3 w% in *Chironomus* was statistically significant (ESM Table S2).

### 3.2. Total Fatty Acid Content and Dry Weight

**Figure 3:**
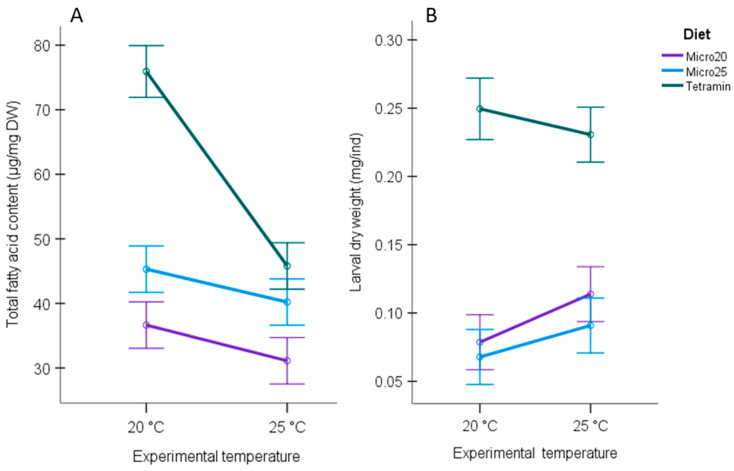
Estimated marginal means for (**A**) Total fatty acid content (µg mg^−1^) in *Chironomus* larvae, and (**B**) Larval dry weight (mg ind ^−1^). Error bars represent 95% confidence intervals. Warming significantly decreased the larval total fatty acid content in the *Microcystis* treatments and in the TetraMin^®^ control (*p* < 0.05). Warming increased the larval dry weight in the *Microcystis* treatment, albeit the increase was statistically significant only for *Micro20* diet. Warming did not affect larval dry weight in the TetraMin^®^ control.

The total fatty acid content and dry weight in *Chironomus* were the highest in larvae fed with TetraMin^®^ (Figure 3A,B). Warming significantly decreased the total fatty acid content from 75.9 to 45.8 µg mg^−1^ DW when larvae were fed with TetraMin^®^. The total fatty acid content in *Chironomus* decreased also in the *Microcystis* diet treatments (Figure 3A, ESM Table S3), but the decline was not as large as for the TetraMin^®^ diet. The larval dry weight (mg ind^−1^) did not respond to increasing temperature in *Chironomus* fed with TetraMin^®^ but increased in *Chironomus* fed with *Microcystis*, albeit the increase was statistically significant only for the *Micro20* diet (Figure 3B, ESM Table S4).

## 4. Discussion

Our results indicated that *Chironomus* desaturated cyanobacterial n-3 and n-6 C_20_ PUFA to physiologically more active forms, i.e., EPA and ARA. This trophic upgrading is ecologically relevant because *Chironomus* are abundant and widespread taxa [18]. The trophic upgrading was dependent on both the abundance of precursors in the diet as well as ambient temperature. Warming decreased the proportions of EPA and ARA in *Chironomus* fed with *Microcystis* indicating decreased bioconversion of C_20_ precursors to EPA and ARA at higher temperatures. This conclusion is supported by the finding that warming did not affect EPA and ARA levels in *Chironomus* fed with TetraMin^®^, which contains EPA and ARA. The proportions of EPA and ARA or their C_20_ precursors in the diet corresponded with the levels of EPA and ARA in *Chironomus*. These results indicate that warming will decrease trophic upgrading of C_20_ precursors but may not significantly affect the direct trophic transfer of algal EPA and ARA.

Compared to vertebrates, knowledge on the enzymes linked to fatty acid desaturation and elongation processes in invertebrates is still fragmented. Kabeya et al. [32] showed that, contrary to vertebrates, many invertebrates possess the genetic code for the de novo synthesis of n-3 and n-6 PUFA, although it is yet to be determined how efficiently these genes are expressed and which factors control the gene expression. Detailed information on desaturase activities in chironomids is incomplete but most likely it includes the traditional ‘Δ6 desaturation—elongation—Δ5 desaturation’ pathway of 18:3n-3 and 18:2n-6 to EPA or ARA, respectively (Figure 1) [25,26]. Results from our previous study indicated that *Chironomus* efficiently Δ5-desaturate dietary C_20_ precursors 20:4n-3 and 20:3n-6 to EPA and ARA, respectively [16]. Results from the current study suggest that the Δ5-desaturation of dietary C_20_ precursors in *Chironomus* is downregulated at higher temperatures. This conclusion is supported by the absence of warming-induced decrease of EPA w% and ARA w% in *Chironomus* when these fatty acids were directly available in the diet, i.e., TetraMin^®^ diet. Note that warming decreased ARA w% in both *Microcystis* diets even if the proportion of 20:3n-6 increased in the *Micro25* diet, suggesting that increased dietary availability of 20:3n-6 did not compensate for the decreased Δ5-desaturation activity. Decreased Δ5-desaturation activity at higher temperatures has been previously documented for crustaceans [28]. Theoretically, also 20:3n-3 may serve as a precursor for EPA if *Chironomus* expresses Δ8-desaturase activity: Δ8-desaturation of dietary 20:3n-3 to 20:4n-3 and subsequent Δ5-desaturation of 20:4n-3 to EPA (Figure 1) [25]. To our knowledge Δ8-desaturase activity has not been confirmed in chironomids, even if this metabolic pathway is theoretically possible and also confirmed in certain teleost fish and molluscs [25,27].

Warming significantly altered the PUFA composition in *Microcystis*, specifically, the n-3/n-6 ratio and the proportion of n-3 C_20_ precursors decreased. The warming-induced decrease in the n-3/n-6 ratio in *Microcystis* is in accordance with previous studies on temperature effects on the fatty acid composition in cyanobacteria and algae [10,11]. The decrease in the n-3/n-6 ratio is most likely due to downregulated *ω3* desaturase expression at higher temperatures as noted before for the cyanobacterium *Synechocystis* sp. (Figure 1) [33]. The *ω6* desaturase was also downregulated at higher temperatures, albeit not as much as *ω3* desaturase [33]. Thus, differing responses of *ω3* and *ω6* desaturase activities to increasing temperature may explain the decline in the n-3/n-6 ratio in *Microcystis*. Low n-3/n-6 ratio and decreased proportion of n-3 C_20_ precursors in *Microcystis* likely reduce membrane lipid damage and help to maintain the function of the photosystem II complex and the electron transport system at higher temperatures [11,34].

Global climate change is predicted to increase surface water temperatures in summer and strengthen the thermal stratification in lakes [35], which in turn have been noted to increase the frequency, intensity, and duration of cyanobacteria blooms [36]. In general, cyanobacteria have a lower n-3/n-6 ratio than eukaryotic algae and most cyanobacteria genera do not produce C_20_ PUFA, thus cyanobacteria blooms have been suggested to decrease the basal production of physiologically important PUFA, such as EPA [6]. *Microcystis* is one of the most common bloom-forming cyanobacteria [4], and the desaturation of C_20_ precursors in *Chironomus* may indicate a higher-than-expected availability of EPA and ARA to upper trophic level consumers in lakes exposed to nontoxic *Microcystis* blooms. However, warming may counteract this phenomenon by decreasing the proportion of n-3 C_20_ precursors in *Microcystis* and downregulating the bioconversion of both n-3 and n-6 C_20_ precursors in *Chironomus*, thus reducing the overall trophic transfer of EPA and ARA in freshwater food webs.

Climate-induced increase in cyanobacteria biomass and the direct temperature effects on the cyanobacterial fatty acids together with the decreased trophic upgrading of C_20_ precursors in chironomids may have cascading effects throughout the food web. The estimated decrease in algal production of EPA and DHA, due to cyanobacteria dominance, correlated with lower levels of EPA and DHA in fish [6]. *Chironomus* contains only trace amounts of DHA even if abundant in the diet [16,24]. Thus, it is highly unlikely that chironomids are important vectors for the trophic transfer of DHA. However, because chironomids are widespread and abundant taxa, they may be highly important for the trophic transfer of EPA in the littoral zone as well as in the pelagic. Chironomid pupae have been noted to be important prey for pelagic fish, thus coupling benthic and pelagic food webs [37]. Additionally, the aerial adult stages serve as vectors for the transfer of EPA from the aquatic environment to the terrestrial consumers, e.g., to insectivore birds and terrestrial spiders [38,39].

The significantly higher larval weights in the TetraMin^®^ control treatment indicate that the *Microcystis* diet was suboptimal for growth. Warming significantly increased larval weight only in the *Micro20* treatment, although an increasing trend was observed also for the *Micro25* treatment. We do not have data on larval length or developmental stages, which have been previously shown to respond to temperature change [20,21,22]. Interestingly, warming significantly decreased the total FA content in the TetraMin^®^ treatment even if we did not observe any effect on the dry weight. Females have higher lipid reserves than males [40]. We did not determine the sex of larvae, but it is unlikely that sex ratios would be systematically biased only in this one treatment. Decreased fatty acid content at higher temperatures was observed also for the *Microcystis* treatments, but the decline was not as large. Lower fat reserves at higher temperatures may be linked with increased physical activity, respiration, and/or different developmental phases as fatty acids are oxidised for energy during molting and chitin synthesis [40].

## 5. Conclusions

The proportions of EPA and ARA in *Chironomus* were strongly dependent on the dietary availability of these PUFA or their C_20_ precursors. Warming decreased the proportion of n-3 C_20_ precursors and n-3/n-6 ratio in *Microcystis*. Additionally, warming decreased the desaturation of C_20_ precursors, resulting in lower levels of EPA and ARA in *Chironomus* fed with *Microcystis*. However, warming did not affect the proportions of EPA and ARA in *Chironomus* if these PUFA were directly available in the diet (TetraMin^®^ fish flakes). These findings indicate that in chironomids the remodeling of PUFA at higher temperatures may occur via downregulated desaturase activity. Furthermore, our results indicate that global warming and eutrophication of lakes and rivers may decrease the n-3/n-6 ratio at the base of the food web as well as the trophic transfer of EPA and ARA to secondary consumers.

## Figures and Tables

**Table 1:** Major fatty acids and sum of n-3 and n-6 PUFA expressed as w% of total fatty acids, and the n-3/n-6 ratio in diets: *Micro20*, *Micro25*, and TetraMin^®^ fish flakes (number of samples *n* = 3 for all diets). The presented fatty acids accounted for more than 99% of all fatty acids in *Microcystis* diets (*Micro20* and *Micro25*), and more than 92% of all fatty acid in TetraMin^®^. The lower percentage in TetraMin^®^ is due to the exclusion of long-chain saturated (≥C_20_ LC-SFA) and monounsaturated fatty acids (≥C_20_ LC-MUFA) from the table, which are prevalent in TetraMin^®^ but not in *Microcystis*, see also Strandberg et al. [16].

Fatty Acid	*Micro20*		*Micro25*		TetraMin^®^	
	Mean	SD	Mean	SD	Mean	SD
14:0	0.40	0.10	0.56	0.16	2.82	0.00
i15:0	0.28	0.06	0.87	0.23	0.05	0.00
15:0	0.08	0.03	0.29	0.08	0.21	0.01
16:0	44.19	1.40	46.23	3.93	15.08	0.40
16:1n-9	0.47	0.02	0.24	0.06	0.08	0.01
16:1n-7	1.40	0.13	1.64	0.09	2.63	0.01
18:0	4.25	1.86	6.38	3.31	11.75	0.17
18:1n-9	1.20	0.13	1.92	0.39	24.86	0.25
18:1n-7	3.00	0.80	5.55	0.70	2.40	0.04
18:2n-6	3.04	0.13	6.79	0.31	22.52	0.19
18:3n-6	9.40	0.35	16.95	1.13	0.01	0.00
18:3n-3	10.87	0.44	5.02	0.92	3.46	0.09
18:4n-3	18.04	1.02	6.05	1.15	0.34	0.02
20:2n-6	0.00	0.00	0.00	0.00	0.19	0.03
20:3n-6	0.35	0.20	0.71	0.69	0.01	0.00
20:4n-6	0.00	0.00	0.00	0.00	0.14	0.02
20:3n-3	0.84	0.37	0.19	0.17	0.02	0.02
20:4n-3	1.93	0.50	0.21	0.16	0.09	0.02
20:5n-3	0.00	0.00	0.00	0.00	2.82	0.15
22:5n-3	0.00	0.00	0.00	0.00	0.32	0.03
22:6n-3	0.00	0.00	0.00	0.00	2.92	0.16
Sum n-6 PUFA	12.79	0.18	24.45	0.19	22.87	0.15
Sum n-3 PUFA	31.69	0.61	11.47	2.11	9.96	0.13
n-3/n-6	2.48	0.05	0.47	0.08	0.44	0.01

**Table 2:** Two-way ANOVA summary table for EPA w% and ARA w% in *Chironomus riparius* across diet and temperature treatments.

Variable	Source	df	SS	MS	F	*p*	Effect Size (Eta Squared)
EPA w%	Diet	2	44.218	22.109	91.176	<0.001	0.829
	Temperature	1	1.148	1.148	4.733	0.040	0.022
	Diet × Temperature interaction	2	1.529	0.764	3.152	0.062	0.029
	Within group	23	5.577	0.242			
	Total	28	53.367				
ARA w%	Diet	2	155.027	77.513	120.595	<0.001	0.810
	Temperature	1	12.715	12.715	19.782	<0.001	0.066
	Diet × Temperature interaction	2	6.598	3.299	5.132	0.014	0.034
	Within group	23	14.783	0.643			
	Total	28	191.348				

## Data Availability

Data are available from the authors upon reasonable request.

## Supplementary Materials

The following are available online at https://www.mdpi.com/article/10.3390/biom11091326/s1, Table S1: Pairwise comparisons of ARA w% in *Chironomus*, Table S2: Pairwise comparisons of arcsine square root transformed EPA w% in *Chironomus*, Table S3: Pairwise comparisons of total fatty acid content (µg mg^−1^) in *Chironomus*, Table S4: Pairwise comparisons of the dry weight (mg ind ^−1^) in *Chironomus*.
