# Supplementary material for: Warming Decreases Bioconversion of Polyunsaturated Fatty Acids in Chironomid Larvae Maintained on Cyanobacterium Microcystis
Source: Biomolecules. 2021 Sep 7;11(9):1326. doi: 10.3390/biom11091326 (PMC8471874; doi:10.3390/biom11091326)
Supplement: Supplementary file 1 [file biomolecules-11-01326-s001.zip › biomolecules-1339394-supplementary.pdf]

Electronic Supplementary Material

Warming decreases bioconversion of polyunsaturated fatty acids in chironomid larvae maintained on cyanobacterium *Microcystis*

Ursula Strandberg[1]*, Timo Ilo[1], Jarkko Akkanen[1], Paula Kankaala[1]

[1] University of Eastern Finland, Department of Environmental and Biological Sciences, Joensuu, Finland

*Corresponding author (ursula.strandberg@uef.fi)

Table S1. Pairwise comparisons of ARA w% in *Chironomus* (based on estimated marginal means) fed with *Micro20*, *Micro25* or Tetramin® diet at different experimental temperatures (20 °C and 25 °C). Diets with significant difference between the experimental temperatures are bolded.

Diet	(I) Experimental temperature	(J) Experimental temperature	Mean Difference (I-J)	Std. Error	Sig.[b]	95% Confidence Interval for Difference[b]	
						Lower Bound	Upper Bound
Micro20	**20 °C**	**25 °C**	**2.020**	**0.507**	**0.001**	**0.971**	**3.069**
Micro25	**20 °C**	**25 °C**	**2.019**	**0.507**	**0.001**	**0.970**	**3.068**
Tetramin	20 °C	25 °C	-0.052	0.538	0.924	-1.164	1.061

b. Adjustment for multiple comparisons: LSD (equivalent to no adjustments).

Table S2. Pairwise comparisons of arcsine square root transformed EPA w% in *Chironomus* (based on estimated marginal means) fed with *Micro20*, *Micro25* or Tetramin® diet at different experimental temperatures (20 °C and 25 °C). Diet with significant difference between the experimental temperatures is bolded.

Diet	(I) Experimental temperature	(J) Experimental temperature	Mean Difference (I-J)	Std. Error	Sig.[b]	95% Confidence Interval for Difference[b]	
						Lower Bound	Upper Bound
Micro20	20 °C	25 °C	0.015	0.009	0.098	-0.003	0.033
Micro25	**20 °C**	**25 °C**	**0.027**	**0.009**	**0.005**	**0.009**	**0.045**
Tetramin	20 °C	25 °C	-0.005	0.009	0.594	-0.024	0.014

b. Adjustment for multiple comparisons: LSD (equivalent to no adjustments).

Table S3. Pairwise comparisons of total fatty acid content (μg mg^{-1}) in *Chironomus* (based on estimated marginal means) fed with *Micro20*, *Micro25* or Tetramin® diet at different experimental temperatures (20 °C and 25 °C). Diets with significant difference between the experimental temperatures are bolded.

Diet	(I) Experimental temperature	(J) Experimental temperature	Mean Difference (I-J)	Std. Error	Sig.[b]	95% Confidence Interval for Difference[b]	
						Lower Bound	Upper Bound
Micro20	**20 °C**	**25 °C**	**5.526**	**2.456**	**0.034**	**0.446**	**10.606**
Micro25	**20 °C**	**25 °C**	**5.098**	**2.456**	**0.049**	**0.018**	**10.178**
**Tetramin**	**20 °C**	**25 °C**	**30.128**	**2.604**	**< 0.001**	**24.740**	**35.516**

b. Adjustment for multiple comparisons: LSD (equivalent to no adjustments).

Table S4. Pairwise comparisons of the dry weight (mg ind $^{-1}$) in *Chironomus* (based on estimated marginal means) fed with *Micro20*, *Micro25* or Tetramin® diet at different experimental temperatures (20 °C and 25 °C). Diet with significant difference between the experimental temperatures is bolded.

Diet	(I) Experimental temperature	(J) Experimental temperature	Mean Difference (I-J)	Std. Error	Sig.[b]	95% Confidence Interval for Difference[b]	
						Lower Bound	Upper Bound
Micro20	**20 °C**	**25 °C**	**-0.035**	**0.014**	**0.017**	**-0.064**	**-0.007**
Micro25	20 °C	25 °C	-0.023	0.014	0.108	-0.051	0.005
Tetramin	20 °C	25 °C	0.019	0.015	0.208	-0.049	0.011

b. Adjustment for multiple comparisons: LSD (equivalent to no adjustments).
